# Paying for Express Checkout: Competition and Price Discrimination in Multi-Server Queuing Systems

**DOI:** 10.1371/journal.pone.0092070

**Published:** 2014-03-25

**Authors:** Cary Deck, Erik O. Kimbrough, Steeve Mongrain

**Affiliations:** 1 Department of Economics, University of Arkansas, Fayetteville, Arkansas, United States of America; 2 Department of Economics, Simon Fraser University, Burnaby, British Columbia, Canada; George Mason University, United States of America

## Abstract

We model competition between two firms selling identical goods to customers who arrive in the market stochastically. Shoppers choose where to purchase based upon both price and the time cost associated with waiting for service. One seller provides two separate queues, each with its own server, while the other seller has a single queue and server. We explore the market impact of the multi-server seller engaging in waiting cost-based-price discrimination by charging a premium for express checkout. Specifically, we analyze this situation computationally and through the use of controlled laboratory experiments. We find that this form of price discrimination is harmful to sellers and beneficial to consumers. When the two-queue seller offers express checkout for impatient customers, the single queue seller focuses on the patient shoppers thereby driving down prices and profits while increasing consumer surplus.

## Introduction

In many markets there are more customers desiring attention than can be accommodated at one time, which means that some customers have to wait, be it in line to checkout at the grocery, on hold with technical support, or for a table at a restaurant. If individuals value their time differently, then there is a socially optimal allocation of scarce queue slots, and traditional first come first serve queuing will be inefficient. Imagine a crowded bakery on a Friday morning. For a customer ordering pastries to take to an important business meeting, waiting in line can be very costly. But for a customer on vacation and planning to enjoy a pastry while reading the newspaper and lounging at a table in the bakery, waiting has a relatively low cost. These arguments are not unique to service industries; users of communication and transportation networks also have heterogeneous waiting costs as certain packets of information and cargo are more time-sensitive than others, and thus all queuing systems that ignore preference heterogeneity are inefficient.

In principle, one could introduce market forces to offset these inefficiencies by allowing high time-cost customers to pay for priority service, either by paying a premium to the seller or via transfers to more patient customers further ahead in the line. However, contracting costs can make such systems difficult to implement, and other less efficient mechanisms are often used instead (such as first come first serve at a bakery where people pull a number). Another alternative is the triage mechanism used in emergency rooms based on the severity of each individual case. While few begrudge this practice in the ER, it is doubtful that people would be as accepting of allowing a baker to decide who was served first based upon their opinion of what the person had planned for the rest of the day. At the same time one can imagine the outrage if a poor person died while waiting at the ER for a wealthy person with a minor scrape to be treated because the latter was willing and able to pay more.

In some situations, however, people are able to pay for priority – as in the movies when a patron slips the maître d' some cash in exchange for a quick seating at a premium table. In the naturally occurring world, Six Flags and other theme parks offer multiple levels of passes at premium prices which allow holders to avoid waiting in line, and for nearly double the usual entry price, tourists can skip the queue when going to the top of the Empire State Building in New York City. A more celebrated example is variable priced toll roads, which have become a hot concept for reducing road congestion. Several U.S. metropolitan areas have converted High Occupancy Vehicle (HOV) lanes over to HOT (High Occupancy or Toll) lanes. Toll roads use prices to limit access to the road. On a conventional toll road, there is a single price regardless of current demand, but with a variable priced toll road the price is adjusted to reflect congestion. For example, a 10-mile stretch of the Riverside Freeway (CA 91) in Orange County, California has variable priced toll lanes, with prices ranging from $1.20 off peak to $10.00 at peak, operating alongside multiple free lanes.

In this paper, we employ a computational model and laboratory experiments to explore a similar concept in a retail environment where multi-queue sellers use premium pricing for express checkout. By charging different prices at each queue, retailers can encourage customers to self-sort according to waiting costs and perhaps thereby acquire a greater portion of the available surplus. As opposed to the toll road example, where there is no competition among highways due to the natural monopoly structure of roads, or the entertainment examples, where products are highly differentiated, retail markets are generally competitive markets offering homogenous goods. Thus, when making pricing decisions, retailers must take into account not just the response of their customers, but of their rivals as well. The most closely related theoretical work to ours is [1]–[3] who model duopoly markets with queuing and stochastic arrival of heterogeneous customers where two identical sellers each control a single server and queue. They show that under certain conditions pure-strategy equilibria exist in which identical sellers charge different prices and segment the market according to waiting cost. So far, however, we know of no research looking at price discrimination when firms control multiple queues in a competitive framework.

At first glance, one may think that the provision of high price and low price queues is just another example of vertical product differentiation, but there is an important difference. In a classic problem of vertical product differentiation, quality is either exogenous or fully under the control of the seller. With queuing, product quality, i.e. waiting time, is determined endogenously by the interaction between prices and consumer behavior. Another departure of our work from these previous papers is that while they have focused on steady state outcomes, we focus on profitability over the natural cycle from store opening when all queues are empty through store closing when no new shoppers arrive but those already in line continue to be served. Two reasons motivate such a departure. First, the nature and the properties of the steady state equilibrium are highly sensitive to parameter choices. Second, the natural cycle approach is better suited in making the bridge between the naturally occurring economy and experiment results.

Compared to current retail practices, price based express checkout is a step beyond what many multi-line retailers offer in the form of “express lines” for those purchasing, say, 12 items or less. The logic of limited quantity queues is that shoppers who are in a hurry and only want a few items may be unwilling to go to a large box store for fear of being stuck in long lines behind shoppers with full carts. These impatient customers may instead choose to go to smaller convenience stores and pay higher prices for a quicker shopping experience. Intuitively, price based express checkout could potentially eliminate a small retailer's ability to poach time sensitive customers.

Ultimately, though, our experimental data suggest that allowing a multi-line seller to offer price based express checkout leads to lower overall prices in the environment we study. This outcome is beneficial to consumers and results in lower seller profits, which may help explain why the practice is not common. The next subsection of the paper briefly reviews related literature. Then we present the model that we analyze computationally and describe the design of our experiments. These allow us to compare price-based express checkout and uniform pricing along with two additional treatments – one that offers a more direct test of the one-shot model's predictions and one that serves as a check on subject responsiveness to model parameters. Then we present the behavioral results, and a final section offers concluding remarks.

### Related Literature

[4] provides the first discussion of using prices to regulate the size of queues, and [5] describes optimal pricing strategies for a single seller operating multiple queues and serving otherwise-homogeneous customers that have heterogeneous waiting costs. Similarly, [6] examines single-server queue price discrimination among heterogeneous customers with asymmetric information, and [7] applies the logic of congestion pricing to the allocation of valuable airport landing slots. Experimental study of these issues have been relatively rare, but see [8] and [9] for experimental implementations of a market for queue priority with a single server. In general, previous laboratory work on queuing has focused on the behavior of customers rather than the sellers operating the queues, e.g. [10]–[14]. See also [15] for an experiment on network congestion.

In the model reported in [2], which is most similar to our setup, when asymmetric-price, pure-strategy equilibria exist, there also exists an equilibrium in which sellers charge the same price. To our knowledge, no one has tested their model in an experimental setting; however, this may be partly due to the fact that the differences in prices charged by the two sellers are typically quite small and would be difficult to distinguish from noise empirically. Our environment differs from theirs in that our sellers are not identical. For an extension to a continuum of sellers, see also [16] and [17], whose discussions of queue price competition differ from ours in that buyers' reservation values are assumed to be sufficiently large that no buyers ever choose not to enter a queue in equilibrium. Similarly, in a model with identical customers and general queue structures, but with heterogeneous service rates rather than waiting costs, [18] prove the existence of mixed strategy oligopoly equilibria.

Practically speaking, the success of any new pricing policy depends on how it is perceived by customers [19]. In a well-known example, Coca-Cola developed a vending machine that would set prices based upon outside air temperature, but consumer response was sufficiently negative that the device was never implemented. On the other hand, overnight shipping and one-hour laundry service are competitive industries that charge a premium for fast service; however, it is likely that customers view the price premium for these services as being justified by the additional effort exerted by the seller. How customers might perceive a store offering a premium-priced express checkout line is an interesting question, but beyond the scope of this paper. We do note that there are two ways to present differential pricing to consumers: offering a discount for those who are more flexible and offering express checkout at a premium for those in a hurry. While offering a discount to the patient and charging a premium to the impatient are not distinct from a traditional theoretical perspective, it is easy to imagine that consumers would view the latter as elitist but have a favorable view of the former. A similar psychological effect likely explains why universities often promote their use of merit-based scholarships, but never advertise that they charge weak students a higher price even though the two statements convey the same meaning.

While it is not always explicitly presented as such, one similar line of research emphasizes the implicit time-cost price discrimination resulting from the practice of offering cents off coupons to customers; only customers with low opportunity costs of time are likely to take the time to cut coupons [20]. See [21] and [22] for a similar argument about rebates; see [23] for a more general discussion of using product differentiation to encourage customer self-sorting; and see [24] for a summary of research on price discrimination in competitive environments. One last important difference between our setting and the rest of the literature on self-sorting and product differentiation is the presence of externalities across consumers. By joining a given line, a consumer imposes a negative externality on other members of the line. A monopoly is able to internalize some of the externality so as to generate higher profits. The presence of competition constrains the ability of firms to internalize this externality. More generally, our paper and this literature can be viewed as part of the evolving focus on customer service in marketing (see [25]) and on multi-attribute competition, in which firms compete for customers on price, service time, quality and so on (see e.g. [26]–[28]).

Finally, our laboratory implementation builds on a rich literature on duopoly (and oligopoly) competition in experimental market settings. [29] summarize the findings from the literature, but to our knowledge no one has studied either competition or price discrimination in laboratory queuing systems.

## Materials and Methods

We address our problem computationally and with controlled laboratory experiments. The following subsections describe the details of our analysis.

### Model and Computational Analysis

Traditionally, multi-queue sellers have set prices that are independent of the checkout line that a shopper uses. However, a seller who operates multiple queues and multiple servers may prefer to charge different prices at each queue to exploit differences in consumers' waiting costs. Shoppers with higher waiting costs would be willing to pay a premium in order to avoid the costs of waiting in line, and thus, the seller can induce consumers to reveal their type by setting different prices and allowing shoppers to self-sort, a form of second degree price discrimination. Therefore, we compare the effects of two policies on prices, profits and consumer welfare. In one case, a multi-queue, multi-server seller sets a single price for all of its queue-server pairs, and in the second case, the seller may engage in time-cost based price discrimination, charging a separate price at each queue; in practice this is equivalent to a situation where one seller operates two retail locations and the other seller operates a single location. The policy question is then whether or not the seller operating two stores sets the same price at both stores.

We construct a model of market competition between two sellers offering identical goods to a population of shoppers, who arrive in the market sequentially. All shoppers want at most one unit of the good and value it equally. Shoppers only differ in their cost of waiting to be served. A seller can serve only one customer at a time for each queue it operates, and the time it takes to service a customer is fixed. Several features of our markets are simplistic. It is unlikely that all consumers have identical values for a product or that service times are identical. However, these realistic complications are not critical to the aspect of the market that is under investigation. Moreover, our model and experimental/computational environment provide a foundation for testing future hypotheses about the impact of these variables.

Shoppers enter queues optimally to maximize their surplus. That is, a shopper compares the value of the item to the total cost of making a purchase, which includes the price and the time cost of waiting to be served. If no queue offers a positive surplus to the shopper, then she balks and does not make a purchase. If some queue offers a weakly positive surplus to the shopper, then she will join the queue offering her the greatest surplus, with any tie broken randomly. Recent technological advances, such as smart phones, make acquiring such information more feasible than it may at first appear, e.g. one can currently search online for real-time information on areas of traffic congestion in most major cities. With the explosion of mobile computing and data sharing the ability to access such information will be enhanced. Under this setup, customers never renege because waiting times are deterministic, and thus if it was optimal to join the line, it is never optimal to leave it.

Formally, we consider a duopoly situation in which one seller controls two queues and the other controls a single line. Both sellers privately and independently set their market prices, which are fixed for N periods. Let 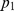 be the price charged by the seller with a single checkout line and let 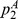 and 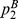 be the prices charged by the seller operating two lines. Traditional uniform pricing by the two-line seller is captured by the constraint that 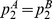, while price based express checkout allows for 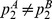. Without loss of generality, we assume that 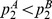.

The number of arriving shoppers in each period is governed by a truncated Poisson 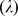 process. The actual distribution was an approximation to a Poisson distribution with an upper bound on the number that could be drawn. This was done to facilitate participant comprehension in the laboratory experiments discussed later in the paper. In particular, our truncated distribution can be described visually as shown in Appendix A in File S1. The computational results are essentially unchanged if one uses a true Poisson distribution. We normalize the service rate so that it takes one period to serve one customer. Each shopper values the good at (has a maximum willingness to pay of) 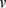. A fraction 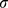 of shoppers are impatient and incur a high cost 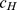 for each period spent waiting to be served. Patient shoppers incur a low cost 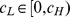 while waiting. Given the normalization that one shopper is served at each queue in each period, a shopper's total wait cost is proportional to the number of people in line ahead of her when she joins the line. Three further clarifications are necessary. First, when the first period begins, there are no shoppers already in line. This mimics a store opening its doors in the morning. Second, while multiple customers can arrive in one time interval, the order in which they consider joining a line is determined randomly. This is consistent with the interpretation of the Poisson distribution as governing the number of occurrences of a random process that occur within a fixed period of time. Third, although there are only N periods, any shopper in line at the end of period N will eventually be served. This final point is similar to a store locking its doors at closing time but allowing those already inside the store to complete their purchases. A similar rule has been employed in other experimental studies of duopoly competition with sequential arrival of buyers (e.g. [30]).

Obviously, the numeric results will depend upon the specific parameter values that are considered. With no particular industry or previous research upon which to base the choice of values, any selection is admittedly somewhat arbitrary. For our simulations and subsequent laboratory experiments, we implement the following parameters:

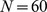

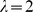

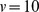

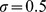

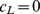

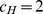

The values of 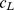 and 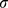 were set for simplicity in both the simulations and the market experiments; 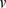 and 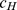 only matter in relation to each other and 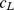. We set N with an eye towards the experiments. In particular, each period lasts one second and thus N = 60 corresponds to a minute in real time. 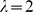 is chosen so that a queue will build if there is a unique low price seller but there is excess service capacity in the market.

To explore optimal behavior, we perform a grid search over 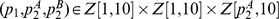 to compute empirical best response functions for each seller. The grid reflects the fact that sellers were required to set integer prices and were not allowed to set prices at 0; nor were they allowed to set prices above the maximum willingness to pay of the buyers as prices equal to 0 or greater than 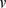 yield zero profit. For each possible price triple, we simulate 2000 realizations of 60 periods and calculate the average profit to each seller.

**Figure 1:**
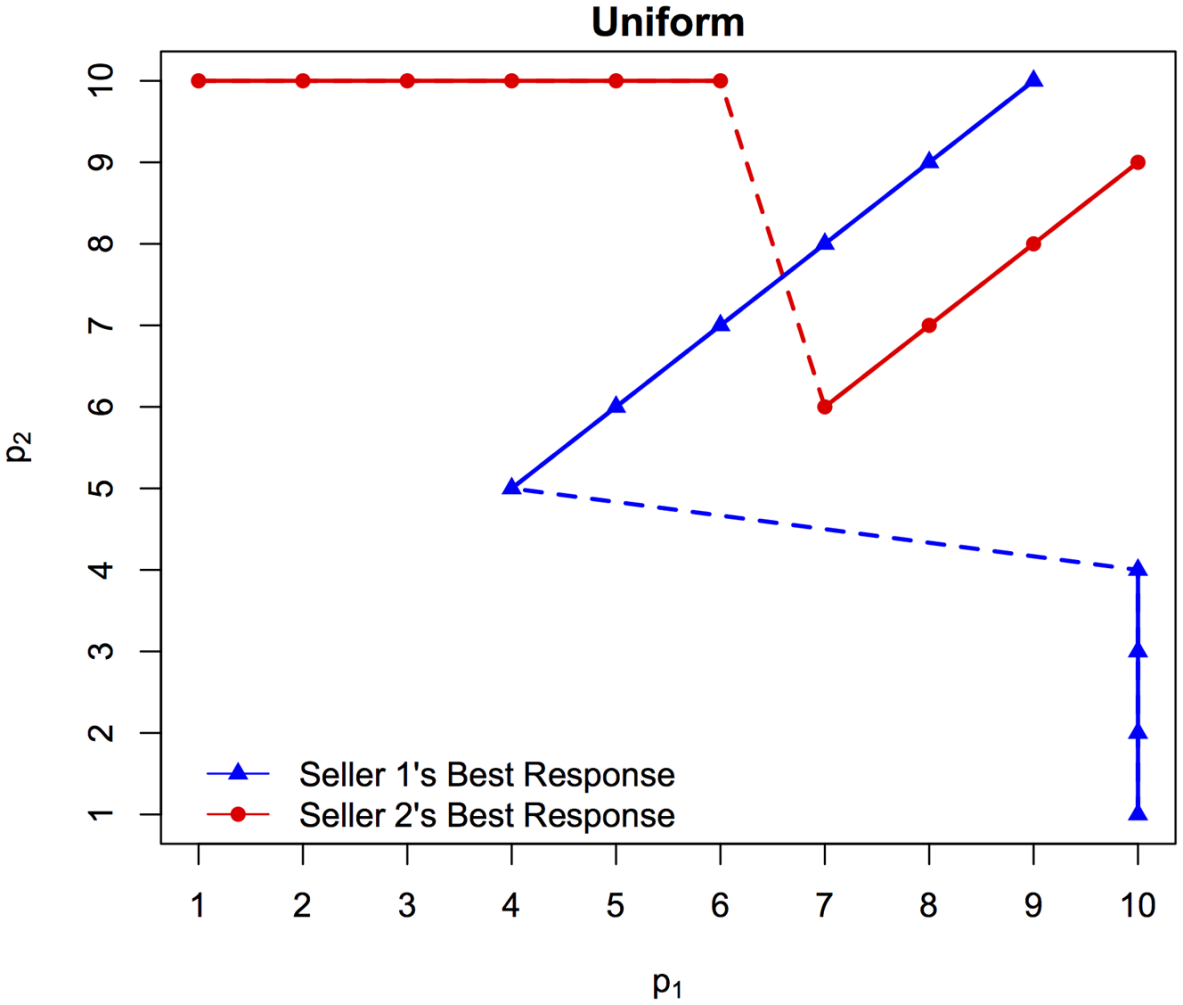
Best Response Functions when Express Checkout Premium is Not Possible.

Figure 1 shows the best response curves for each seller with uniform pricing. Notice that both sellers have a one-dimensional best response given that each sets a single price. In a strict sense, as only integer prices are allowed (both in the simulation and the accompanying experiment), the best responses are the markers that appear in Figure 1. The lines are added to help visualize the relationships. Solid lines denote a continuation of a strategy (to undercut or poach) whereas dashed lines indicate a change in strategy. In the uniform pricing case, there is no pure strategy equilibrium (i.e. the best response curves do not coincide). Both sellers prefer to undercut their rival by one unless their rival is charging a sufficiently low price in which case it is optimal to set a price of 10 and poach impatient shoppers. As shown in Figure 1, there is an asymmetry in how low a seller is willing to price, with the single-line seller willing to price as low as 5 and the two-line seller only willing to go as low as 7. To determine the Nash equilibrium of this game, we first eliminate all of the dominated pure strategies (ex: the single line seller setting a price less than 5). Given this reduced game it is then straightforward to calculate the mixed strategy Nash equilibrium. The result is that the single line seller should set a price of 5 with approximately probability 0.06, a price of 7 with probability 0.90, and a price of 9 with probability 0.04. The two-line seller should charge a price of 6 on both lines with approximately probability 0.49, a price of 8 on both lines with probability 0.19, and a price of 10 with probability 0.32. Table 1 summarizes pricing under the mixed strategy Nash equilibrium. In equilibrium, the two-line seller would expect to earn approximately $451 for each 60-period market day.

**Table 1:** Mixed Strategy Nash Equilibrium Prices by Treatment.

		Price		
		(Probability)		
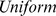	One-Line Seller	5	7	9
		(0.06)	(0.90)	(0.04)
	Two-Line Seller	6, 6	8, 8	10, 10
		(0.49)	(0.19)	(0.32)
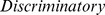	One-Line Seller	4	6	8
		(0.62)	(0.30)	(0.08)
	Two-Line Seller	5, 8	7, 8	10, 10
		(0.60)	(0.23)	(0.17)

When the two-line seller can price discriminate, that seller has a best response surface, which would result in a three-dimensional figure. In general, the single-line seller prefers to undercut its rival unless the minimum price offered by the rival is very low, as with uniform pricing. The two-line seller's best response is generally to undercut the single-line seller's price with one line and charge a premium at the other line. As in the uniform pricing case, there is no pure strategy equilibrium. While the size and nature of the reduced game is more complicated in this case, one can again calculate the mixed strategy Nash equilibrium treating each price pair the two-line seller could charge as a strategy. To solve for the equilibrium we used Gambit [31]. In equilibrium, the single-line firm will set its price at 4, 6, and 8 with probabilities of approximately 0.62, 0.30, and 0.08, respectively. The two-line seller will set price pairs of (5,8), (7,8), and (10,10) with probabilities of approximately 0.60, 0.23, and 0.17 respectively; see Table 1 for a summary. In equilibrium, the two-line seller would expect to earn approximately $447, or 1% less than when price discrimination is not possible, and this is driven by the lower average prices charged in the discriminatory case. Thus, it appears that rather than being profitable, the ability to price discriminate by offering premium express checkout actually harms the two-line seller, albeit minimally. Why, then, does the two-line seller not simply set the same price at both lines? This is because he always has an incentive to use one line to undercut the price set by the one-line seller, ensuring that he can sell to all the patient shoppers while still exploiting some impatient customers with the higher priced line. Without the ability to price discriminate the two-line seller is forced to do one or the other.

To check the robustness of our analysis, we replicate the simulations with 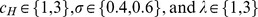, varying each parameter in isolation. The basic results are similar, and we find no pure strategy equilibria under any of these parameterizations. However, it is worth noting that if the proportion of shoppers who are impatient (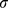) is sufficiently low, the situation reduces to Bertrand competition.

Finally, for completeness we point out that, with our baseline parameters, a three-line monopolist forced to set a uniform price would optimally charge 10 at each line, and a three-line firm that was allowed to set different prices at each line would have one line priced at 9 and two priced at 10. This simple change would increase monopoly profit by over 18% because it reduces balking by impatient shoppers. This latter outcome is the joint profit-maximizing collusive strategy, which can be achieved in two ways under Discriminatory, 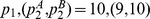 and 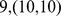, and in only one way under Uniform, 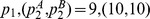.

### Experimental Design and Procedures

#### Ethics Statement

This study was conducted with the approval of the Institutional Review Board of the University of Arkansas. All experiments were conducted with the informed consent of 48 healthy adult subjects who were free to withdraw from participation at any time. Only individuals who voluntarily entered the experiment recruiting database were invited, and informed consent was obtained in writing prior to the beginning of each experimental session.

To further explore the impact of sellers charging for express checkout, we turn to a two-treatment, between subjects controlled laboratory experiment. In the experiment, human subjects participated as sellers while buyers were operationalized as truthfully revealing robots. This approach is justified because we study a setting in which buyers do not have market power and do not make repeated purchasing decisions [32]. Subjects were randomly assigned to be either a one-line seller or a two-line seller and retained that role throughout the experiment. The two main treatments are distinguished by whether the two-line seller is forced to charge a single price for both of his queues (Uniform treatment) or not (Discriminatory treatment). Subjects were randomly and anonymously matched with a seller in the other role and interacted with that person for the entire session which consisted of 40 markets, each lasting N = 60 periods. Twelve subjects were in the laboratory at one time so that no one could identify who their rival was. The treatment was fixed for the entire session. The fixed matching procedure and N were common information, but the number of markets was not. The subjects also had complete information regarding the parameters presented in the previous section. As argued in [33], maintaining fixed matching more closely replicates naturally occurring markets, as few sellers ever interact with their rival only once. Given our interest in the applied problem, we chose to implement a fixed matching protocol for our main experiment. As described later, we also examine a random re-matching protocol, which is more appropriate for a strict test of the one-shot model.

**Figure 2:**
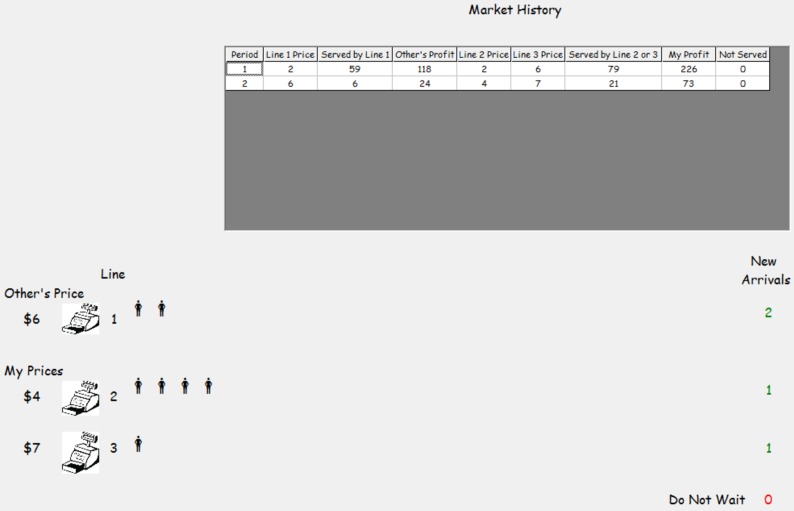
Subject Seller Interface.

Subject sellers set their prices at the beginning of a market. Each period was one second and therefore a market lasted just over one minute since any customers already in line at the end of the 60th period continued to be served at the rate of one per second. Once prices were set, both sellers could observe current prices and were shown buyers arriving in the market. Figure 2 shows the screen interface. A buyer in line was represented by a stick figure. Those buyers standing beside the image of a cash register are in the queue, and thus sellers could determine how long each line was at any point. How many shoppers arrived each period, how many joined each queue, and how many shoppers balked was displayed on the lower right of the subject screen. Also available to subject sellers was a historical record that showed previous prices and earnings by market. Sellers intentionally were not informed of the wait cost of a particular shopper because this information is not available to sellers in naturally occurring retail markets. To reduce variation across sessions, a single set of approximately 4800 realizations (40 markets×60 periods per market×an expectation of 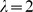 buyers per period) of buyers was used in every duopoly. This practice helps ensure that differences between sessions are generated by the treatments and endogenous differences in pricing behavior.

We conducted two additional experiments to provide further insight into these markets. The first of these, called the Random treatment, replicates the Discriminatory experiment except that sellers are randomly and anonymously re-matched after each 60 period market, though subjects retain their roles as either one-line or two-line sellers throughout. As mentioned above, this procedure offers a cleaner test of the mixed strategy Nash equilibrium, which is developed as a one shot game ignoring any strategic implications of current behavior on future outcomes. In particular, repetition allows for reputation building and the threat of future punishment of non-cooperative behavior. The folk theorem implies that any set of strategies that generates profits for both players in excess of the one shot Nash equilibrium can also be supported as a Nash equilibrium in the repeated game when players have sufficiently low discount rates. In our setting this means that Discriminatory should lead to prices weakly between the one-shot equilibrium level and the monopoly level. Since the ability to punish non-cooperative behavior in the future is eliminated in Random, behavior is hypothesized to coincide with the one shot Nash equilibrium. Thus, prices in Discriminatory are hypothesized to be weakly greater than those in Random.

The second additional experiment, called the Patient treatment, also replicates the Discriminatory treatment except that all customers are patient (i.e. 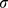 = 0) and so will always simply join the lowest priced line. Casual introspection yields that Bertrand competition should drive all prices down to their minimum (1) in the one shot game and that the monopoly outcome is to charge a price of 10 at every line. Thus, with the repeated play (fixed pairings) employed in the Patient treatment, the folk theorem implies that any price could be charged by at all three lines in equilibrium. Still, it seems reasonable to suppose that the elimination of impatient customers will lead to lower prices. Thus, this treatment is designed to verify that our sellers are responsive to shopper characteristics.

The experiments were conducted in the Behavioral Business Research Laboratory at the University of Arkansas. Participants were recruited randomly from the lab's database of undergraduate volunteers. A common, but nave, criticism of laboratory experiments that involve undergraduates is that the subjects lack the sophistication necessary to understand the problem. First, theoretical models such as the one developed here are silent as to the identity of the decision-maker and only specify that it is attempting to maximize its profit. Second, this critique wrongly assumes that it is the absolute level of behavior that is relevant and not the comparative static effect between treatments. It is true that a laboratory market will not generate the same prices as another market (naturally occurring or inside the lab) with different parameters. It is also true that knowing the price in a naturally occurring market, such as the one for consumer package goods, will not inform you of the price for other items such as clothes. Unless the sophistication and experience of the decision maker is expected to have differential effect across treatments, then the comparative static effects are robust to such changes. Finally, when researchers have explicitly compared sophisticated subjects and undergraduates, the behavioral differences are often quite small (see for example, [34] studying asset market trading, [35] in common value auctions outside the familiar domain of the experts, and [36] for myopic loss aversion among traders).

Six distinct seller pairs competed in each of the Uniform, Discriminatory, and Patient treatments. An additional twelve subjects participated in the Random treatment. On average, sessions lasted one hour. Each subject received a payment of $5 for arriving to the session on time. Subjects were also paid based upon their profits in the experiment. All prices, costs, and values in the market were denoted in Lab Dollars, which were converted into USD at the end of the experiment at a rate of 150 Lab Dollars = 1 USD for two-line sellers and 100 Lab Dollars = 1 USD for one-line sellers. Average salient earnings in the experiment were $9.68 with a high of $20.25 and a low of $4.00, not including the arrival payment. File S1 contains the experiment instructions and a comprehension quiz administered to ensure participants understood the instructions.

## Results

Table 2 provides summary statistics for all four treatments. The results are presented in four subsections. The first two focus on the main treatment comparison – Discriminatory vs Uniform. In the first subsection we focus on the aggregate effects of the treatment variable while the second subsection examines role-specific behavior. The third subsection reports the results of the Random treatment and compares those to both the theoretical prediction and the observed behavior in Discriminatory. The fourth subsection reports the results of the Patient treatment.

**Table 2:** Average Per Period Summary Statistics, by Treatment, markets 21–40.

	(1)	(2)	(3)	(4)
	*Uniform*	*Discriminatory*	*Random*	*Patient*
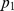	6.55	4.88	3.89	3.65
	(1.97)	(1.97)	(1.50)	(2.49)
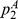	7.21	4.78	3.52	3.95
	(2.07)	(1.85)	(1.40)	(2.42)
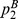	–	6.50	4.85	4.49
		(1.60)	(1.53)	(2.88)
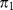	300.29	213.16	145.69	181.45
	(142.59)	(90.67)	(63.68)	(203.20)
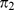	488.08	364.79	283.19	209.94
	(152.78)	(142.46)	(80.67)	(240.21)
Price Paid	6.66	4.83	3.64	3.25
	(1.90)	(1.69)	(0.87)	(2.06)
Buyer Surplus	533.83	732.94	886.35	972.13
	(226.22)	(196.94)	(104.62)	(265.16)
Number Balking	1.34	0.37	0.06	NA
	(2.50)	(1.56)	(0.28)	

Standard deviations in parentheses.

### Aggregate Treatment Effects between Discriminatory and Uniform

We find that when multi-line sellers are prohibited from engaging in price based express checkout, prices and profits are higher than when sellers can engage in this form of price discrimination. This result is despite the fact that the multi-line seller could opt to not engage in price discrimination by simply setting a single price at both lines. Further, consumer welfare is greater when the two-queue sellers offer price based express checkout. The buyer result is not tautologically equivalent to the seller result, because higher prices can reduce queue length and thus wait costs. Below, we provide details on these two main findings.

FINDING 1: Price based express checkout lowers market prices and reduces seller profits.

**Figure 3:**
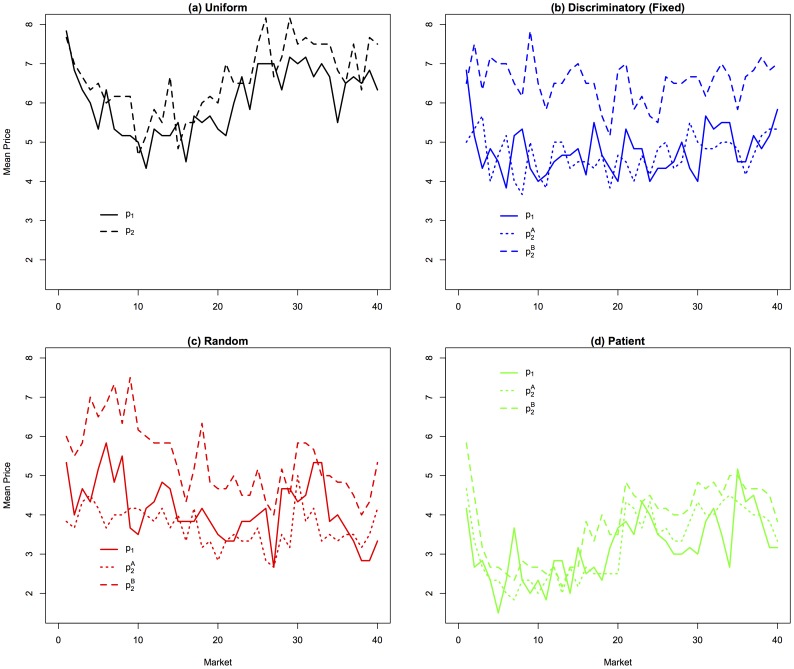
Time Series of Prices by Treatment. Averaged across sessions.

Evidence: Figure 3 displays time series (market periods 1–40) of mean prices charged by queue, averaged across all duopolies in each treatment. We observe notable changes in pricing behavior over time in the first half of the experiment before behavior stabilizes over the last half, so our analysis hereafter focuses exclusively on the last half of the experiment. Our main conclusions comparing the Uniform and Discriminatory treatments are robust to using the entire dataset, but in comparing the Discriminatory treatment to the Patient and Random treatments, the early price variation displayed in panels (c) and (d) of Figure 3 leads to qualitatively different conclusions than those we reach when we restrict attention to the last 20 periods. We will discuss these differences in more detail below. Clearly evident from the two top panels of the figure is the fact that mean prices charged, computed over all three lines, are higher in the Uniform treatment than in the Discriminatory treatment (6.99 vs. 5.39, respectively). Moreover, mean profits are 394 Lab Dollars/market in the Uniform treatment and 289 Lab Dollars/market in the Discriminatory treatment. These comparisons are supported by panel regressions reported in Table 3.

**Table 3:** GLS Random Effects Regressions Showing Mean Session-Level Treatment Effects, 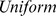 vs. 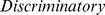, markets 21–40.

	(1)	(2)	(3)	(4)	(5)
	Price Charged	Profit	Price Paid	Buyer Surplus	# Balking
*Discriminatory*	−1.603**	−210.425**	−1.828**	199.117**	−0.975
	(0.734)	(93.286)	(0.828)	(90.892)	(0.747)
Constant	6.989***	788.375***	6.662***	533.825***	1.342**
	(0.562)	(64.200)	(0.589)	(66.745)	(0.682)
Observations	240	240	240	240	240
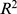	0.182	0.207	0.207	0.182	0.0524

Standard errors in parentheses, clustered at the pair level.

Statistical significance designated as follows:

*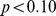,

**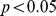,

***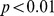.

Columns (1) and (2) report regression analysis where the dependent variables are the mean price charged by sellers and the total profit, respectively, in each market of each session. In both specifications, as well as the other three discussed below, the independent variables are a constant term and a dummy variable that takes a value of 1 in the Discriminatory treatment and 0 otherwise. We include random effects for each session to control for repeated measures and report heteroskedasticity robust standard errors. We employ random effects rather than fixed effects because we observe each pair in only one treatment. In columns (1) and (2), the estimated coefficients of the Discriminatory treatment dummy variable are both negative and significant (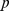-values
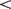
0.05) indicating that the ability to implement a premium express checkout policy is harmful to sellers.

We now turn to the buyers' side of the market where we find that price based express checkout is beneficial to consumers.

FINDING 2: Consumer welfare is higher with price based express checkout.

Evidence: As with Finding 1, we report summary statistics and estimate treatment effects using panel regressions that account for the repeated measures nature of our data. In Finding 1, we computed the mean price charged by sellers, but to understand the effects on consumer welfare, one should compute the mean price paid by buyers, which will differ because buyers sort according to their waiting costs and will buy from the lowest priced seller ceteris paribus. Over the second half of the experiment, markets 21–40, the mean price paid is higher in the Uniform treatment than the Discriminatory treatment (6.66 vs. 4.83). Similarly, consumer surplus is higher in the Discriminatory treatment than in the Uniform treatment (733 vs. 534 Lab Dollars/market).

Columns (3) and (4) of Table 3 report panel regression analysis in support of the evidence presented above. Column (5) looks for evidence that price based express checkout benefits consumers by reducing balking. In columns (3)–(5) the dependent variables are the mean price paid, total buyer surplus, and the number of buyers that balk in each market of each session, respectively. Again, we include session-specific random effects, and we estimate heteroskedasticity robust standard errors. A negative and significant estimated coefficient on the treatment dummy in equation (3) supports the claim that consumers pay lower prices in the Discriminatory treatment, and a positive and significant coefficient in equation (4) indicates that buyers earn more surplus (both 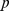-values
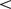
0.05). An insignificant estimated coefficient of the treatment dummy in column (5) indicates no statistically significant difference in balking when discrimination is possible. Taken together, the evidence strongly supports the claim that buyers are better off under a regime of price based express checkout.

While price discrimination is typically understood to result in a net transfer of surplus from buyers to sellers, we observe the opposite effect due to the competitive pressure applied by the one-line seller on the two-line seller's lower priced queue.

### Firm-Type Specific Treatment Effects between Discriminatory and Uniform

Why does the ability to engage in price discrimination harm sellers? The answer can be found in the top two panels of Figure 3. Observe that in the Uniform treatment, the two-line seller sets very high prices and those prices are higher than the price set by the one-line seller. Recall that the monopoly price, that is the price that would be charged if one seller operated all three queues, is 10, the same as the buyer's value. In such a situation, a price cut by the two-line seller has a large negative impact on its profitability because it is no longer able to charge impatient people a high price. By contrast, if the two-line seller can engage in price discrimination, then it can lower one of its prices in an attempt to take market share from the one-line seller while maintaining a high price at its second queue to continue exploiting impatient shoppers. This is exactly the pattern that is observed in the top right hand panel of Figure 3, where the single-queue seller is competing heavily with the lower-priced of the two-queue seller's lines. As a result of the competition, the premium price at the two-line seller is reduced too.

Not being able to discriminate increases the cost of competing for patient consumers by aggressively lowering prices. A uniform price acts as a disciplining device. Behaviorally, if the two-line seller could credibly commit not to engage in price discrimination then it would prefer to do so, thereby raising profits; however, for whatever price the single-line seller charges, the two-line seller would prefer to engage in price discrimination. This phenomenon is similar to the temptation to defect in a prisoner's dilemma game despite the fact that cooperation is jointly preferred. The tendency of sellers to engage in tacit collusion is a well-known finding in repeated duopoly games (See e.g. [29], [37], [38]); however, in our experiment if firms collude, whichever firm is able to charge a price of 9 will serve the bulk of the customers and thus earn the lion's share of the profits and the two lines setting a price of 10 will attract only impatient customers when the low price queue is long. So the incentives to undercut are strong in this environment. This deterrent to collusion is amplified in the Discriminatory treatment where there is a coordination problem to determine which firm will charge the lower price. Higher prices in the Uniform treatment provide evidence that cooperation may be simpler the fewer variables that must be coordinated. That is, it may be easier for the two sellers to collude when there are only two prices instead of three.

To verify this type specific pattern statistically, we estimate the difference in behavior between the Uniform and Discriminatory treatments. Separate linear panel regressions are conducted for each seller type. For one-line sellers, we estimate a single model where the dependent variable is the price charged by seller 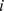 in market 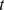. For the two-line firms, we estimate three different models: in the first, the dependent variable is the mean price charged over both lines; in the second, the dependent variable is the lower of the two prices charged; and in the third, the dependent variable is the higher of the two prices. In the Uniform treatment, the higher and lower price are the same, so these will allow us to estimate whether the two-line Uniform sellers price closer to the high or low price charged by Discriminatory sellers. In all of the models the independent variables are a Discriminatory treatment dummy variable and a constant term. We include random effects for each seller to control for repeated measurements and we estimate heteroskedasticity robust standard errors.

Table 4 reports the regression output. In the regression comparing one-line sellers reported in column (1), a negative and significant estimated coefficient on the Discriminatory treatment dummy indicates that one-line firms charge significantly higher prices in the Uniform treatment. Comparing two-line sellers, column (2) reports that mean prices are lower in the Discriminatory treatment, and columns (3) and (4) indicate that this effect is primarily driven by the fact that prices are competed down significantly at the lower priced of the two lines.

**Table 4:** GLS Random Effects Regressions Showing Firm-Level Treatment Effects, 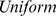 vs. 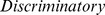, markets 21–40.

	(1)	(2)	(3)	(4)
	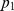	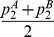	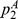	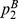
*Discriminatory*	−1.675**	−1.567**	−2.425***	−0.050
	(0.814)	(0.732)	(0.843)	(0.730)
Constant	6.550***	7.208***	7.208***	6.550***
	(0.606)	(0.560)	(0.560)	(0.606)
Observations	240	240	240	240
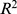	0.154	0.155	0.277	0.0002

Standard errors in parentheses, clustered at the pair level.

Statistical significance designated as follows:

*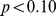,

**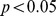,

***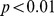.

### Testing the Theoretical Predictions with the Random Treatment

While it is an equilibrium of the repeated game in the Discriminatory treatment for the sellers to play the one-shot mixed strategy Nash equilibrium in each stage game, the Folk theorem implies there are other mutually beneficial (for the sellers) pricing strategies that could be sustained in equilibrium. Thus, the Random treatment, which eliminates the repeated play aspect of the game, is a cleaner test for equilibrium behavior and prices are expected to be weakly lower in Random as compared to Discriminatory.

To investigate these two comparisons, we estimate a linear panel regression where the dependent variable is the price charged at line 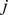 by seller 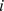 in market 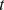 and the independent variables are a constant term, a dummy variable for the Random treatment, a dummy variable that takes a value of 1 if the price was set at a two-line seller's higher priced line and 0 otherwise, a similar dummy for the lower priced line of a two line seller, and interaction terms between the Random treatment and the higher price and lower price dummy variables. Thus, the baseline case captured by the constant term is the price of the single-line seller in the Discriminatory treatment. We include random effects for each seller to control for repeated measurements, and we estimate heteroskedasticity robust standard errors.

Table 5 reports the regression output for comparing the fixed and random rematching protocols when premium express checkout is possible. The estimated coefficient on the Random treatment dummy is negative and marginally significant. This provides some evidence that repeated interactions can lead to higher prices in this setting, although we also note that prices remain far below the collusive level even with repeated play. The interactions between Random and the dummies for High and Low Price lines are not statistically different from 0 indicating that relative prices are not impacted by repeated play, which casts further doubt on the idea that sellers are colluding in Discriminatory.

**Table 5:** GLS Random Effects Regressions Showing Firm-Level Price Comparisons, *Discriminatory (Fixed)* vs. *Random*, markets 21–40.

	(1)
	Price Charged
*Random*	−0.983*
	(0.569)
High Price Line	1.625**
	(0.665)
Low Price Line	−0.092
	(0.816)
*Random*×High	−0.667
	(0.759)
*Random*×Low	−0.283
	(0.875)
Constant	4.875***
	(0.533)
Observations	720
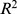	0.247

Standard errors in parentheses, clustered by firm.

Statistical significance designated as follows:

*(*p*<0.10),

**(*p*<0.05),

***(*p*<0.01).

We now turn to the question of how well the mixed strategy equilibrium of our model predicts behavior in this situation. The evidence suggests that neither the Random nor the Discriminatory treatment exhibits prices consistent with the theoretical predictions. Specifically, the expected price at each line in equilibrium based upon the mixing distribution presented above is 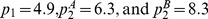. In the Discriminatory (Fixed) treatment the average observed prices were 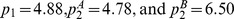 and in the Random treatment they were 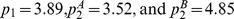. For completeness, average prices in the Uniform treatment were 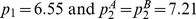. A Wald test rejects the joint hypothesis that the observed prices match the equilibrium predictions in both the Discriminatory and Random treatments (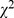 = 20.74 and 198.74, respectively, 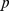-values
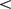
0.01). As compared to the theoretical prediction, it appears the sellers are too competitive, a result similar to [39] that also uses posted offer market experiments to explore pricing strategies in retail markets.

To test whether the finding above is driven by outliers and to determine how many individual sellers chose prices consistent with the mixed-strategy Nash equilibrium (MSNE) over the last 20 markets, we estimate a separate OLS regression for each seller in the Discriminatory and Random treatments where the dependent variable is price charged and the independent variable(s) are (1) for one-line sellers, a constant term, and (2) for two-line sellers, a constant term and a high-priced line dummy variable. After each regression, we perform F-tests: (1) of the hypothesis that for one line sellers that the estimated coefficient on the constant term is equal to 4.9, and (2) of the joint hypothesis for two-line sellers that the estimated coefficient on the constant term is equal to 6.3 and the sum of the estimated coefficients on the constant term and the high-priced dummy is equal to 8.3. If we are able to reject the null hypothesis here, we have evidence that an individual seller charged a mean price that was inconsistent with the mixed strategy; the data indicate that only 3/12 sellers in the Discriminatory treatment and 1/12 sellers in the Random treatment charged prices consistent with the MSNE, suggesting that deviation is systematic and not driven by outliers. An analogous analysis indicates that 3/12 sellers in the Uniform treatment follow the MSNE there.

### Results of Manipulation Check – Patient Treatment

In the Patient treatment, all buyers simply select the lowest price line, no one will balk, and line length is irrelevant. This essentially creates Bertrand competition and all prices should be set to one in the one shot game. Playing the one-shot Nash equilibrium leads to very low profits for both sellers. The repeated nature of interaction supports other mutually beneficial price combinations, so while one would expect lower prices in the Patient treatment than in the Discriminatory treatment due to the change in buyer values, the returns to successful collusion also differ. To compare behavior between treatments, we estimate a linear panel regression where the dependent variable is the price charged at line 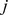 by seller 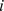. The independent variables are as in the regression reported in the previous subsection except that the dummy variable for the Random treatment is replaced by a dummy variable for the Patient treatment. Again, the baseline for comparison captured by the constant term is the price of a single line seller in the Discriminatory treatment. We include random effects for each seller to control for repeated measurements and we cluster standard errors at the session level (i.e. at the level of each seller-pair).

The estimation results are reported in Table 6. The coefficients for the Patient treatment dummy variable and the interaction terms involving the Patient treatment are statistically insignificant. This appears to suggest that the manipulation check was unsuccessful. However, inspection of Figure 3 tells a different story. Over the first few periods, subjects in Patient quickly compete prices down close to the theoretical prediction resulting in low profits. After experiencing this outcome many subjects begin to actively collude and raise their prices.

**Table 6:** GLS Random Effects Regressions Showing Firm-Level Price Comparisons, *Discriminatory (Fixed)* vs. *Patient*, markets 21–40 in Col. 1 and markets 1–20 in Col. 2.

	(1)	(2)
	Price Charged	Price Charged
*Patient*	−1.225	−2.033***
	(0.905)	(0.710)
High Price Line	1.625**	1.900***
	(0.665)	(0.488)
Low Price Line	−0.092	−0.125
	(0.816)	(0.669)
*Patient*×High	−0.783	−1.425
	(1.405)	(0.898)
*Patient*×Low	0.392	−0.017
	(1.325)	(0.975)
Constant	4.875***	4.692***
	(0.533)	(0.450)
Observations	720	720
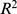	0.143	0.390

Standard errors in parentheses, clustered by firm.

Statistical significance designated as follows:

*(*p*<0.10),

**(*p*<0.05),

***(*p*<0.01).

**Figure 4:**
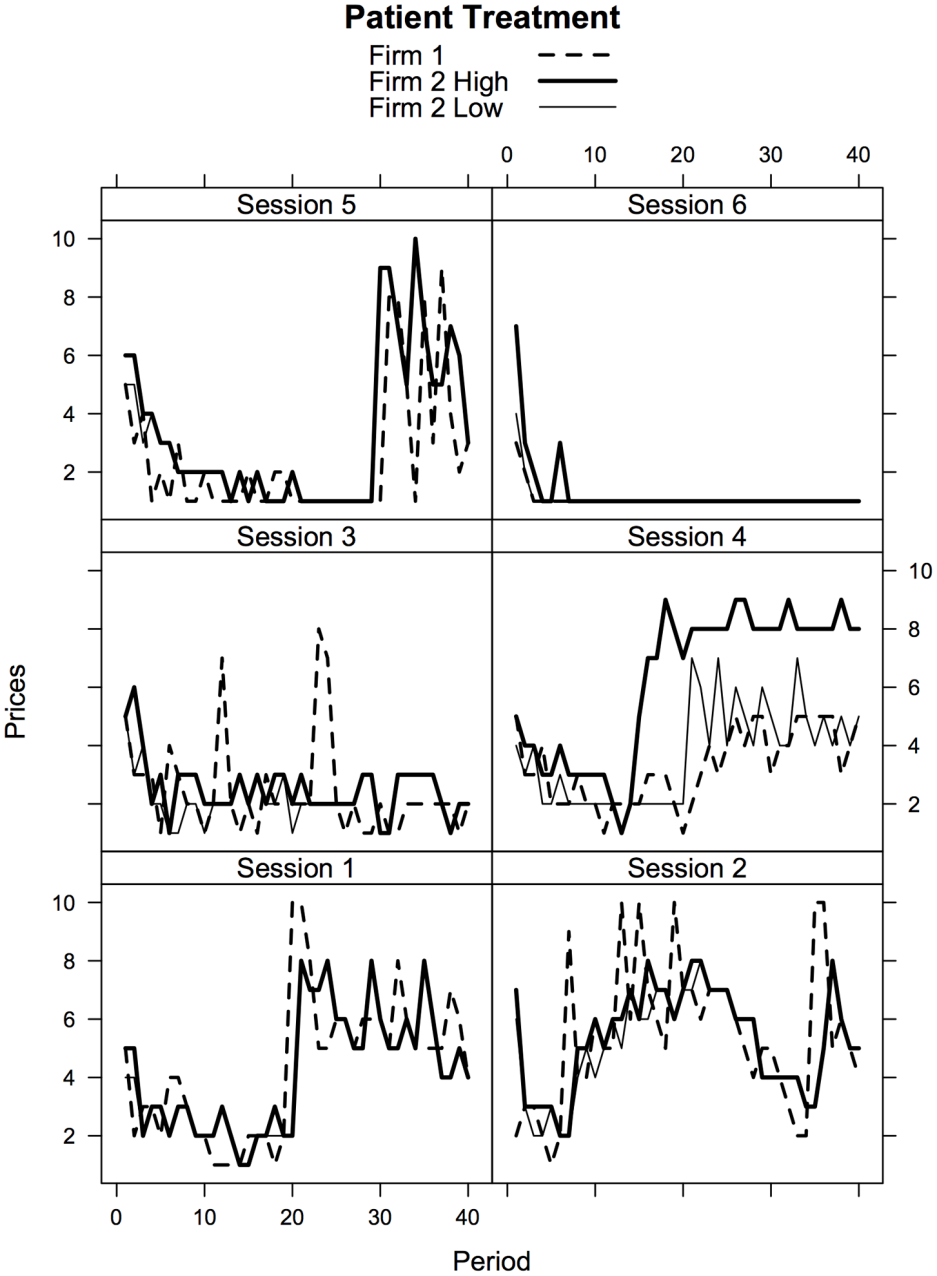
Time Series of Prices by Session, 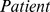 treatment.

Figure 4 shows the behavior of each pair in the Patient treatment. In session 1, after gradually falling prices and profits for the first half of the experiment, the single line seller unilaterally raises his price leading the two-line firm to follow. Sessions 2, 4, and 5 show similar patterns while sessions 3 and 6 show competitive behavior with a few price spikes that can be interpreted as failed attempts to encourage collusion.

If one reruns the estimation comparing the Patient and Discriminatory treatments using data from market periods 1–20 instead of the second half of the experiment, the results are very different (see Table 6). In the early periods, prices are indeed lower on average in the Patient treatment, consistent with the comparative statics predictions of the model. This result indicates that subjects are reacting as expected to the customer characteristics in this market and that sellers understand the environment. The observed behavior is in line with previous Bertrand competition experiments, which have found that sellers are typically competitive, but rarely so competitive that they push prices (and profits) all the way down to the predicted level (see the discussion of posted offer market experiments in [29]).

## Discussion

When we began this project, we were struck by the puzzling rarity of time-cost based price discrimination in situations where customers have to physically queue for service in competitive markets. Exceptions, such as priority boarding for travelers on an airline, prove the rule since many of these customers have earned their status by repeatedly flying on a single airline and essentially giving the chosen airline market power in exchange for priority service. [40] argue that price dispersion in the airline industry may be partly driven by frequent flyer programs. See [41] for an empirical estimate of the switching costs imposed by frequent flyer programs. The examples given earlier of HOT lanes and Six Flags passes are from essentially monopoly markets. There are also some seemingly forgone discrimination opportunities among monopoly providers. For example, some spectators at a ballgame (ourselves included) would likely be willing to pay an even higher price for beer if it meant avoiding a long line in the concourse while missing the game. We do not normally observe restaurants (formally) allowing customers to pay for priority seating or mass retailers operating more than twenty registers offering a faster checkout line at a higher price. This form of time based price discrimination is distinct from package shippers, film developers, and dry cleaners offering faster completion times for a higher price, as these services can be viewed as different goods and the process occurs after the transaction.

One initially plausible reason for the absence of time-cost price discrimination is that people might be offended by such practices (for a general discussion of customer acceptance of various polices see e.g. [19]). Interestingly, [42], who interviewed two hundred firms in relation to their pricing practices, reveals that the most frequent reason for keeping price stable was to avoid “antagonizing or causing difficulties to customers”. However, as the toll road and amusement park examples suggest, such moral concerns are not always a deterrent to discriminatory pricing. Moreover, customer distaste would likely be reduced or eliminated as shoppers came to realize they were made better off by the practice at exactly the point at which it is most important to them, i.e. when their time is most valuable.

Our experimental findings suggest an alternative reason why time cost price discrimination, such as paying a premium for express checkout, is rare: when sellers are in direct competition for customers, queue price discrimination can be harmful to the sellers and industries that employ it. This finding is similar to that in [43] who find that coupon-based price discrimination can be harmful to firms in oligopolistic industries and to [44] who find that price discrimination can lead to lower prices and profits in markets with switching costs. Specifically, we find that average posted prices are lower when the multi-line seller cannot engage in price discrimination. This change is relatively small theoretically, but substantial behaviorally. As a result of the changing posted prices associated with allowing wait cost based price discrimination by the multi-line seller, average paid prices fall, reducing profits and increasing consumer surplus. Uniform pricing across queues by the multi-line seller appears to be more profitable when there is a mix of patient and impatient customers because it forces the multi-line seller to abandon the impatient customers when pursuing the patient ones. In contrast, with discrimination the multi-line seller can attempt to capture both markets, which leads the single-line rival to price more aggressively. This drives down the price for patient shoppers, which in turn limits the premium that impatient customers are forced to pay. As predicted by the model and demonstrated in a separate experiment, when all customers are patient the marketplace becomes very competitive and prices fall, at least initially.

Our main findings are based upon the situation where rivals interact repeatedly for an indefinite horizon. While this is more reflective of the naturally occurring world, the threat of future punishment can theoretically support cooperation. Thus, we conducted an additional experiment where each interaction was one-shot in order to give the model its best shot. We find only marginal evidence of a difference between the repeated game and the one-shot game. This suggests that collusion may not be as problematic in more complicated market games as it is in simpler duopoly games or prisoner's dilemma games more generally (see [29] for a discussion). Added complexity may also explain why collusion is rarely observed in repeated games with more than three players (see for example [38].

Our work contributes to the already rich literature on multi-attribute competition in which sellers compete on both price and service time (see Related Literature above). At the same time, we believe that our work suggests several important new directions for research in retail economics. For example, we treat the number of queues that each seller operates as exogenous, but this decision is ultimately endogenous and can be varied with the expected demand such as when a large grocery hires fewer cashiers for the late night shift. Another extension that advances in technology will likely facilitate is the ability to dynamically adjust the price of express checkout with queue length, something akin to the toll pricing on CA 91.

## Supporting Information

File S1 **Appendix A Experiment Instructions and Appendix B Comprehension quiz.**
(DOCX)
