# Supplementary material for: Paying for Express Checkout: Competition and Price Discrimination in Multi-Server Queuing Systems
Source: PLoS One. 2014 Mar 25;9(3):e92070. doi: 10.1371/journal.pone.0092070 (PMC3965397; doi:10.1371/journal.pone.0092070)
Supplement: File S1 — Appendix A Experiment Instructions and Appendix B Comprehension quiz. (DOCX) [file pone.0092070.s001.docx]

**Supplementary Appendix A: Experiment Instructions (Discriminatory Treatment)**

*Two-Line Seller*

You are participating in a study about economic decision making. You will be paid in part based upon your decisions, so it is important that you understand the instructions completely. If at any point you have a question, please raise your hand. Please do not communicate with anyone else during this study.

You will be in the role of a firm. You will be randomly matched with another person in the study who will also be in the role of a firm. Neither of you will know the identity of the person you are matched with, but you will remain matched with this other firm for the entire study.

The two firms interact in a market. Both firms set prices and then buyers decide if they want to make a purchase or not. Buyers only want *at most* one unit and are willing to pay up to $10 for it if they do not have to wait. Therefore, sellers cannot charge more than $10. It takes a firm 1 second to serve a buyer in a check-out line. Half of the buyers incur no wait cost; however, the other half of the buyers are impatient and incur a $2 cost for each second they spend waiting in line to check-out. This means that the most an impatient buyer will pay is $8 if there is already 1 buyer in the line, $6 if there are 2 buyers in the line, $4 if there are 3 buyers in the line, and $2 if there are 4 people in line. Since a seller cannot set a price at or below $0 an impatient buyer will never join a line that already has 5 or more people in it. Notice that a buyer does not incur any cost while checking-out, only while waiting in line for others to check out. For simplicity, sellers can only whole dollar prices.

In this experiment, the computer will act as the buyers. Each computer buyer will look at the prices and wait times to determine whether or not to make a purchase. Each computer buyer will always take its best deal (buy from the firm offering it the smallest total cost, which is price + wait cost) assuming that the cost is not more than $10. The computer buyer will break any tie between its best deals randomly. If the total cost is greater than $10, the buyer will exit the market without buying.

The experiment proceeds in a series of periods. At the start of each period you and the other firm will set prices. After that, some number of buyers will arrive every second for 60 seconds. The number that arrive each second follows a particular *Poisson* distribution with an average arrival rate of 2 potential buyers per period, which means that the chance that:

0 potential buyers arrive is 0.14 or 14%
1 potential buyer arrives is 0.27 or 27%
2 potential buyers arrive is 0.27 or 27%
3 potential buyers arrive is 0.18 or 18%
4 potential buyers arrive is 0.09 or 9%
5 potential buyers arrive is 0.04 or 4%
6 potential buyers arrive is 0.01 or 1%

You can visualize this as a bucket with 100 balls in it. 14 of the balls are labeled “0”, 27 are labeled “1”, 27 are labeled “2”, 18 are labeled “3”, 9 are labeled “4”, 4 are labeled “5”, and 1 is labeled “6”. Each second one of these balls is drawn out, the number on the ball determines how many customers arrive that second, and then the ball is placed back in the bucket. This is shown graphically below.

🄌🄌🄌🄌🄌🄌🄌🄌🄌🄌🄌🄌🄌🄌

➊➊➊➊➊➊➊➊➊➊➊➊➊➊➊➊➊➊➊➊➊➊➊➊➊➊➊

➋➋➋➋➋➋➋➋➋➋➋➋➋➋➋➋➋➋➋➋➋➋➋➋➋➋➋

➌➌➌➌➌➌➌➌➌➌➌➌➌➌➌➌➌➌

➍➍➍➍➍➍➍➍➍

➎➎➎➎

➏

As buyers show up, they will make their decisions and get in a line if they decide to make a purchase. The choices of the buyers who arrive each second will be shown on your screen under the label “New Arrivals.” Each person in line at any point in time will be shown on your screen as a. Notice that new arrivals have to wait in line behind everyone who is already in line. Buyers who chose not to buy are labeled “Not Served.” After all of the buyers who arrive in the 60 second period have finished shopping, both you and the other firm will have the opportunity to change your prices and the whole process will repeat.

Your firm will operate two check-out lines and can charge different prices for each line. The other firm will only operate a single check-out line. This will not change for the entire study.

A table on your screen will record prices each period along with the number of buyers served and profits.

After the study is complete, you will be paid based upon your total profit. Your profit each period is simply the price you charge times the number of buyers you serve (you do not have any costs). Your total profit is simply the sum of your profit each period. The amount that you will be paid in cash is your total profit / 1500 = $US payment.

*One-Line Seller*

You are participating in a study about economic decision making. You will be paid in part based upon your decisions, so it is important that you understand the instructions completely. If at any point you have a question, please raise your hand. Please do not communicate with anyone else during this study.

You will be in the role of a firm. You will be randomly matched with another person in the study who will also be in the role of a firm. Neither of you will know the identity of the person you are matched with, but you will remain matched with this other firm for the entire study.

The two firms interact in a market. Both firms set prices and then buyers decide if they want to make a purchase or not. Buyers only want *at most* one unit and are willing to pay up to $10 for it if they do not have to wait. Therefore, sellers cannot charge more than $10. It takes a firm 1 second to serve a buyer in a check-out line. Half of the buyers incur no wait cost; however, the other half of the buyers are impatient and incur a $2 cost for each second they spend waiting in line to check-out. This means that the most an impatient buyer will pay is $8 if there is already 1 buyer in the line, $6 if there are 2 buyers in the line, $4 if there are 3 buyers in the line, and $2 if there are 4 people in line. Since a seller cannot set a price at or below $0 an impatient buyer will never join a line that already has 5 or more people in it. Notice that a buyer does not incur any cost while checking-out, only while waiting in line for others to check out. For simplicity, sellers can only whole dollar prices.

In this experiment, the computer will act as the buyers. Each computer buyer will look at the prices and wait times to determine whether or not to make a purchase. Each computer buyer will always take its best deal (buy from the firm offering it the smallest total cost, which is price + wait cost) assuming that the cost is not more than $10. The computer buyer will break any tie between its best deals randomly. If the total cost is greater than $10, the buyer will exit the market without buying.

The experiment proceeds in a series of periods. At the start of each period you and the other firm will set prices. After that, some number of buyers will arrive every second for 60 seconds. The number that arrive each second follows a particular *Poisson* distribution with an average arrival rate of 2 potential buyers per period, which means that the chance that:

0 potential buyers arrive is 0.14 or 14%
1 potential buyer arrives is 0.27 or 27%
2 potential buyers arrive is 0.27 or 27%
3 potential buyers arrive is 0.18 or 18%
4 potential buyers arrive is 0.09 or 9%
5 potential buyers arrive is 0.04 or 4%
6 potential buyers arrive is 0.01 or 1%

You can visualize this as a bucket with 100 balls in it. 14 of the balls are labeled “0”, 27 are labeled “1”, 27 are labeled “2”, 18 are labeled “3”, 9 are labeled “4”, 4 are labeled “5”, and 1 is labeled “6”. Each second one of these balls is drawn out, the number on the ball determines how many customers arrive that second, and then the ball is placed back in the bucket. This is shown graphically below.

🄌🄌🄌🄌🄌🄌🄌🄌🄌🄌🄌🄌🄌🄌

➊➊➊➊➊➊➊➊➊➊➊➊➊➊➊➊➊➊➊➊➊➊➊➊➊➊➊

➋➋➋➋➋➋➋➋➋➋➋➋➋➋➋➋➋➋➋➋➋➋➋➋➋➋➋

➌➌➌➌➌➌➌➌➌➌➌➌➌➌➌➌➌➌

➍➍➍➍➍➍➍➍➍

➎➎➎➎

➏

As buyers show up, they will make their decisions and get in a line if they decide to make a purchase. The choices of the buyers who arrive each second will be shown on your screen under the label “New Arrivals.” Each person in line at any point in time will be shown on your screen as a. Notice that new arrivals have to wait in line behind everyone who is already in line. Buyers who chose not to buy are labeled “Not Served.” After all of the buyers who arrive in the 60 second period have finished shopping, both you and the other firm will have the opportunity to change your prices and the whole process will repeat.

Your firm will only operate a single check-out line. The other firm will operate two check-out lines and can charge different prices for each line. This will not change for the entire study.

A table on your screen will record prices each period along with the number of buyers served and profits.

After the study is complete, you will be paid based upon your total profit. Your profit each period is simply the price you charge times the number of buyers you serve (you do not have any costs). Your total profit is simply the sum of your profit each period. The amount that you will be paid in cash is your total profit / 1000 = $US payment.

**Supplementary Appendix B: Comprehension Quiz**

1. If the experimenter makes 100 draws from the bucket and each time replaces the drawn ball after recording the number written on it, how many of those draws (on average) will be “2” balls?
   1. 14
   2. 27
   3. 9
   4. 50

For the following questions, suppose the market looks as follows:

The price on line1 is $5, and there are 3 buyers in line.

The price on line 2 is $7, and there is 1 buyer in line.

The price on line 3 is $7, and there are 2 buyers in line.

1. If a buyer arrives with a waiting cost of $0/second, which line would the buyer choose to enter?
   1. Line 1
   2. Line 2
   3. Line 3
   4. The buyer wouldn’t enter a line
2. Now suppose the buyer’s cost is $2/second, which line would the buyer choose to enter?
   1. Line 1
   2. Line 2
   3. Line 3
   4. The buyer wouldn’t enter a line
3. True or False: The firm that operates two lines can charge different prices at the two lines. _____________
